# Site-selective local fluorination of graphene induced by focused ion beam irradiation

**DOI:** 10.1038/srep19719

**Published:** 2016-01-29

**Authors:** Hu Li, Lakshya Daukiya, Soumyajyoti Haldar, Andreas Lindblad, Biplab Sanyal, Olle Eriksson, Dominique Aubel, Samar Hajjar-Garreau, Laurent Simon, Klaus Leifer

**Affiliations:** 1 Department of Engineering Sciences, Ångström Laboratory, Uppsala University, Sweden; 2 Institut de Sciences des Matériaux de Mulhouse, UMR 7361-CNRS, UHA, France; 3 Department of Physics and Astronomy, Ångström Laboratory, Uppsala University, Sweden; 4 Department of Chemistry, Ångström Laboratory, Uppsala University, Sweden

## Abstract

The functionalization of graphene remains an important challenge for numerous applications expected by this fascinating material. To keep advantageous properties of graphene after modification or functionalization of its structure, local approaches are a promising road. A novel technique is reported here that allows precise site-selective fluorination of graphene. The basic idea of this approach consists in the local radicalization of graphene by focused ion beam (FIB) irradiation and simultaneous introduction of XeF_2_ gas. A systematic series of experiments were carried out to outline the relation between inserted defect creation and the fluorination process. Based on a subsequent X-ray photoelectron spectroscopy (XPS) analysis, a 6-fold increase of the fluorine concentration on graphene under simultaneous irradiation was observed when compared to fluorination under normal conditions. The fluorine atoms are predominately localized at the defects as indicated from scanning tunneling microscopy (STM). The experimental findings are confirmed by density functional theory which predicts a strong increase of the binding energy of fluorine atoms when bound to the defect sites. The developed technique allows for local fluorination of graphene without using resists and has potential to be a general enabler of site-selective functionalization of graphene using a wide range of gases.

Light and ion beams have been used intensively to enhance the surface functionalization, both for removing and adding atoms^1^,^2^,^3^. In cases where ions are involved, ion implantation turns out to be a direct method for functionalization and is widely achieved for carbon related materials such as carbon nanotubes (CNTs), graphite and fullerenes^4^,^5^,^6^,^7^. An alternative route for functionalizing surfaces is to utilize plasmas containing radicals that interact with a large sample surface directly.

Graphene functionalization is a key enabler to open graphene to applications and has attracted significant interest, because it can alter the chemical, electronic and structural properties of this fascinating material^8^,^9^,^10^,^11^,^12^,^13^,^14^. Using electron beams, fluorine could be locally removed from fluorinated graphene^15^. Plasma enhanced graphene functionalization has been shown in hydrogenation, oxidation and halogenation experiments^16^,^17^,^18^,^19^. In most of these plasma based functionalizations, it is the radicalized gas atom that interacts and reacts with graphene surface. All the methods mentioned above are not site-selective or extra photo/electron resists must be introduced, leading to impurities on the graphene surface, that are difficult to remove.

In this work, we carried out the site-selective fluorination of graphene by using focused ion beam irradiation in XeF_2_ atmosphere in high vacuum. The main idea of this approach is to radicalize the graphene surface locally by utilizing high-energy ion irradiation and, simultaneously, provide a fluorine containing precursor molecule to the carbon atom while it is in the radicalized state. In this method, the fluorine atoms are predominately localized in the nanometer regime surrounding the inserted defects. X-ray photoelectron spectroscopy (XPS), Raman spectroscopy, scanning tunneling microscopy (STM) and density functional theory (DFT) calculations were performed to verify the formation of fluorinated graphene and explain the mechanism of focused ion irradiation induced fluorination.

## Results

### Sample preparation

**Figure 1:**
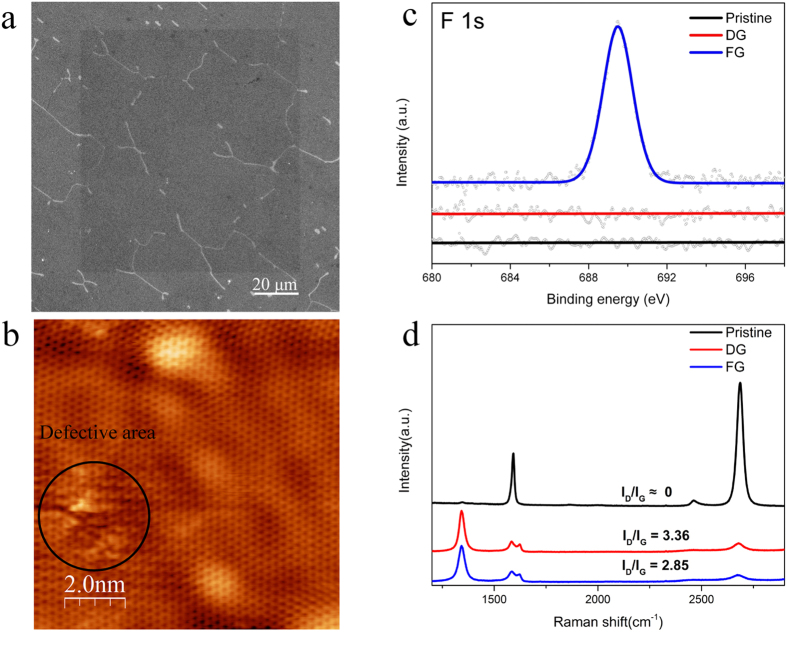
Characterization of pristine graphene, defected graphene (DG) and fluorinated graphene (FG). (**a**) Scanning electron microscope (SEM) image of local functionalization of graphene (100 μm × 100 μm) with ion doses of 10^13^ ions/cm^2^ and simultaneous 167 s gas exposure. (**b**) Scanning tunneling microscopy image of DG under the same ion dosage. (**c**) X-ray photoelectron spectroscopy spectra of F 1 s peak of pristine graphene, DG and FG. FG reveals a distinguished F 1 s peak, and the F 1 s spectrum of pristine graphene as well as DG is given as a reference. (**d**) Raman comparison of pristine graphene, DG and FG. Lower I_D_/I_G_ in FG in contrast to DG indicates lower degree of defects density and larger crystalline size.

The graphene was fluorinated by using ion dosage and XeF_2_ exposure time of 10^13^ ions/cm^2^ and 167 s respectively. A selected irradiation area of 100 × 100 μm^2^ (shown in Fig. 1a) SEM image shows clear the contrast after fluorination. To make a clearly comparison, in the first step, three samples, pristine graphene, defected graphene (DG) and fluorinated graphene (FG), are prepared. DG is prepared by ion irradiation without any gas exposure, while FG is prepared by ion irradiation with simultaneous gas injection.

### Fluorination

The fluorination was performed by a low dosage focused ion beam (30 kV Ga^+^) irradiation on graphene samples in FEI Strata DB235 (FIB/SEM) under high vacuum conditions (5.5 × 10^−5^ mbar), while simultaneously, XeF_2_ was supplied during the irradiation by the gas injection system (GIS) inside FIB/SEM chamber with a partial pressure at the nozzle exit of 600 Pa^20^. The main idea of this fluorination is to, by defect insertion, create carbon dangling bonds that react with fluorine containing molecules within selected area. The advantage of using 30 keV gallium ions is that, after radicalizing the graphene surface, gallium ions penetrate into the substrate and do thus not substitute carbon atoms nor accumulate on graphene surface (see Supporting Information S1).

### STM, XPS and Raman spectroscopy

When high-energy Ga^+^ ions irradiate graphene locally, the defected structure of DG under ion irradiation of 10^13^ ions/cm^2^ shown in Fig. 1b is obtained. By controlling FIB parameters, the selected irradiation area can be localized in variable sizes from micrometers to nanometers. Under this ion irradiation, the graphene maintains most of its perfect lattice shown in Fig. 1b, while the damaged part shows significant defected structures. As for the inserted defect types, they are mainly vacancies with the typical size several angstroms (see Supporting information S2). By a combination of SRIM calculations of 30 keV Ga^+^ ions with experimental correction factor of sputtering yield in graphene^21^, the sputtering yield of carbon in graphene on top of SiO_2_ is 0.3 atoms per ion, meaning that 0.15% of carbon atoms are removed from the graphene lattice at an ion dosage of 10^13^ ions/cm^2^. The XPS spectra (Fig. 1c) display a distinguished fluorine signal in FG, indicating the formation of fluorinated graphene. Taking into account the sensitivity factor (cross section), the transmission function of spectrometer and the mean free path, the atomic ratio of fluorine to carbon gives 3.5 ± 0.4%. If we assume that F atoms are located where a vacancy has been created, then three dangling bonds or less are available for F. If all carbon dangling bonds were saturated by F atoms at vacancy concentration of 0.15%, the expected fluorine concentration would be 0.45%, a factor of 8 different from the experimental result. The possible reason for this is that, by defect insertion, more carbon atoms surrounding defect site are activated and react to XeF_2_, contributing to the increase of fluorine coverage. We also noticed that the F 1 s signal increases for grazing angle which signify that the fluorine species are mainly localized at the surface. Raman spectroscopy is an efficient tool to reveal the structural evolution of graphene^22^,^23^. The intensity of D band (at 1350 cm^−1^), which is nearly negligible in the pristine graphene sample, increases clearly after irradiation. In contrast, the intensity of the 2D band (at 2700 cm^−1^) decreases sharply, which indicates the translational symmetry of the sp^2^ carbon bonds in graphene is broken in both irradiated samples. Compared with DG, the intensity ratio of D and G band (I_D_/I_G_) in FG is significantly lower, which could correspond to a lower degree of structural disorder. From the I_D_/I_G_ ratio that we obtained, an effective crystalline size that is bigger in FG (7 nm) than in DG (5.7 nm)^24^,^25^.

## Discussion

**Figure 2:**
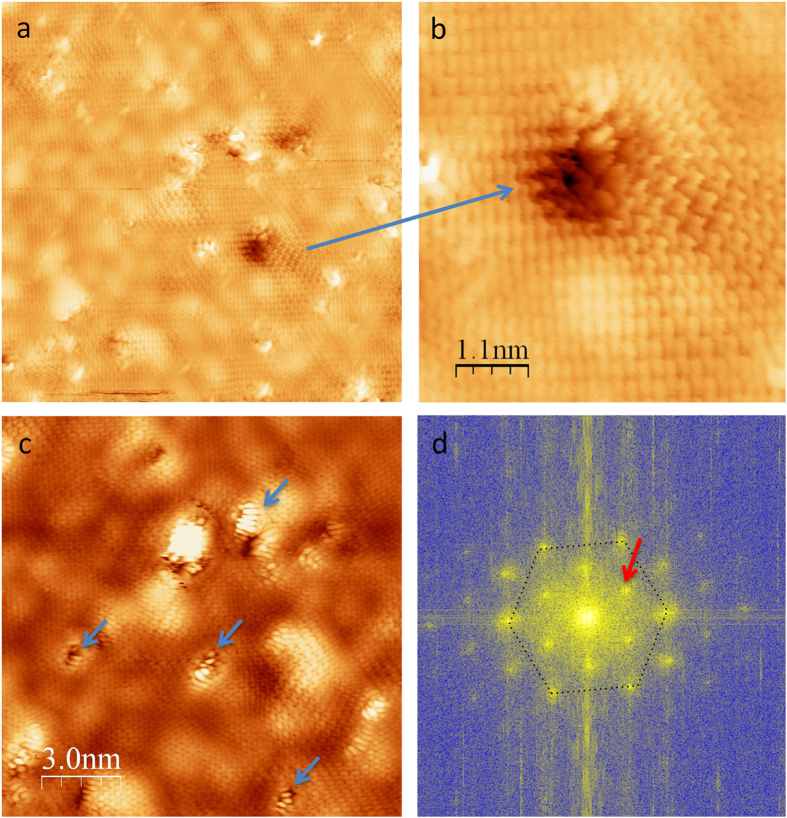
STM images of fluorinated graphene (FG). (**a**) 20 × 20 nm^2^ area, (**b**) Zoom in image of a hole defect showing standing waves pattern, (**c**) Other area 15 × 15 nm^2^ showing bright feature decorating holes (blue arrows) attributed to fluorine atoms. (**d**) shows the FFT (Fast Fourier Transform) of (**a**). This reveals the first Brillouin zone with the hexagonal lattice and K points (red arrows) associated to the standing waves pattern due to intervalley scattering.

To understand the atomic structure further, more STM experiments were performed on FG sample shown in Fig. 2 and all images are taken under low bias voltage (−75 mV) at Fermi level. Figure 2a gives an area of 20 × 20 nm^2^ in fluorinated graphene and the surface is covered by defects, corrugations and bright features. The corrugations correspond to the standing waves with different structure particularly around the defects. The Fast Fourier Transform of Fig. 2a reveals the first Brillouin zone with the hexagonal lattice and K points associated to the standing waves pattern due to intervalley scattering^26^. Standing waves are visible all over the surface and fairly all defects are “connected” by standing waves pattern. Contrarily to point defect which creates standing waves with hexagonal symmetry^27^, larger defects as zoomed in Fig. 2b display standing waves as straight lines similarly to standing waves observed at step edges, meaning that the fluorinated graphene remains metallic^28^. In order to get stable STM images, we have annealed the sample gently in UHV with temperature up to 200 °C. Before and after annealing bright features are always visible and XPS measurements show that fluorine is stable by the annealing (see Supporting information S3). In Fig. 2c, the bright features, which are attributed by fluorine, are much clearer and combing the observation of standing waves associated to delocalized electron along the conjugated sp^2^ bonds in Fig. 2b, it can conclude that the fluorine atoms are localized on the defects created by the irradiation only.

**Figure 3:**
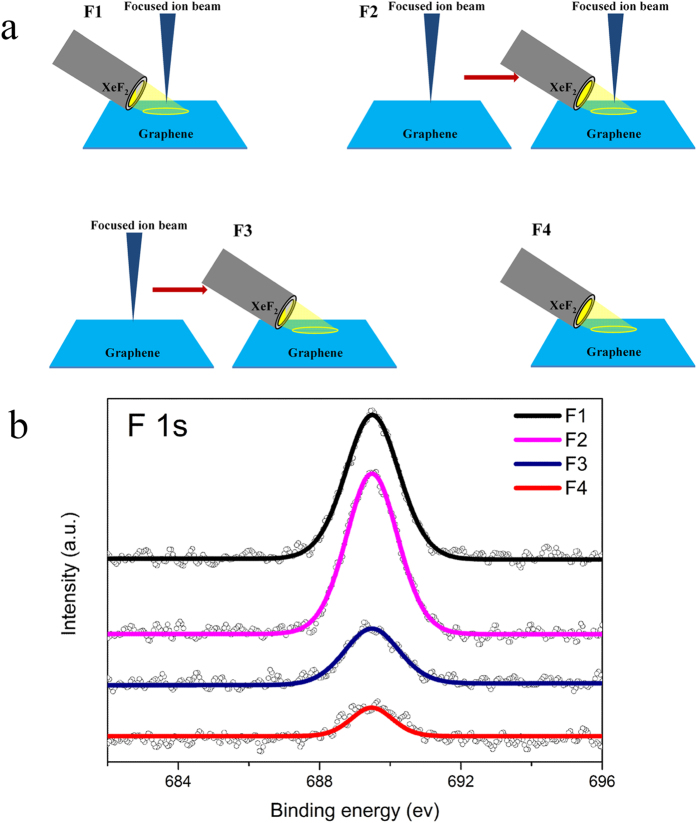
Schematics of comparison experiments and related XPS of F 1 s peaks. The schematics of four different samples are shown in (**a**). F1: ion irradiation accompanied by simultaneous gas exposure (167 s). F2: first ion irradiation and ion irradiation again accompanied by gas exposure (167 s). F3: first ion irradiation and then only gas exposure (167 s). F4: only gas exposure (167 s) as a control experiment. In the schematics, each ion irradiation refers to 10^13^ ions/cm^2^. (**b**) XPS of F 1 s intensity comparison of different samples. Comparing the fluorine intensity of F1-F4, these relations are found: I_F2_ > I_F1_, I_F3_ > I_F4_ and I_F1_ ≈ 6I_F4_. F4 is the controlled experiment to study the influence of XeF_2_ exposure without irradiation.

In order to better understand the mechanism of the fluorination in graphene, more comparison experiments were designed and the schematic details are shown in Fig. 3a. Comparing the fluorine intensity of F1-F4 (Fig. 3b), it is found that the fluorine intensity of F2 (I_F2_) is higher than that of F1 (I_F1_), and I_F1_ is around five times larger than I_F4_. To explain the result, a simple model could be descripted taking sample F1 as an example. In sample F1, when the gas nozzle is open, XeF_2_ will be injected into the vacuum chamber and form a thin film on graphene surface^29^. At the same time, incoming Ga^+^ ions remove carbon atoms from the graphene lattice and then propagate into the substrate deeply, resulting in the local radicalization of graphene surface. The nature of this radicalization is the formation of carbon dangling bonds as well as the activation of carbon atoms surrounding defects. These carbon atoms can efficiently react with XeF_2_. When the ion irradiation and gas exposure are closed simultaneously, the gas film on graphene surface evaporates and the fluorinated graphene remains. Accordingly the study of Utke *et al.*^30^, XeF_2_ will not be decomposed under ion irradiation, which confirms our explanation that the fluorination occurs between defects and XeF_2_. The difference between F1 and F2 is that, before gas exposure, F2 has an extra ion irradiation, implying more defects are formed. The higher fluorine intensity of F2 versus F1 could be explained by the defects created in the first step of F2 being at least partially saturated by fluorine. In contrast to F1, F3 shows lower fluorine intensity which can be explained as the partial saturation of defects by residual gas molecules present in the vacuum chamber within the time gap between ion irradiation and gas exposure. The intensity increase in F3 as compared to F4 equally can be understood by this mechanism. F4 is the control experiment, and the fluorine is mainly from the direct reaction between graphene and XeF_2_^17^. Comparison between F1 and F4 indicates that ion assisted fluorination of graphene is a highly efficient method with the fluorine concentration being a factor of six higher than the one obtained by direct XeF_2_ exposure of graphene^17^,^31^.

**Figure 4:**
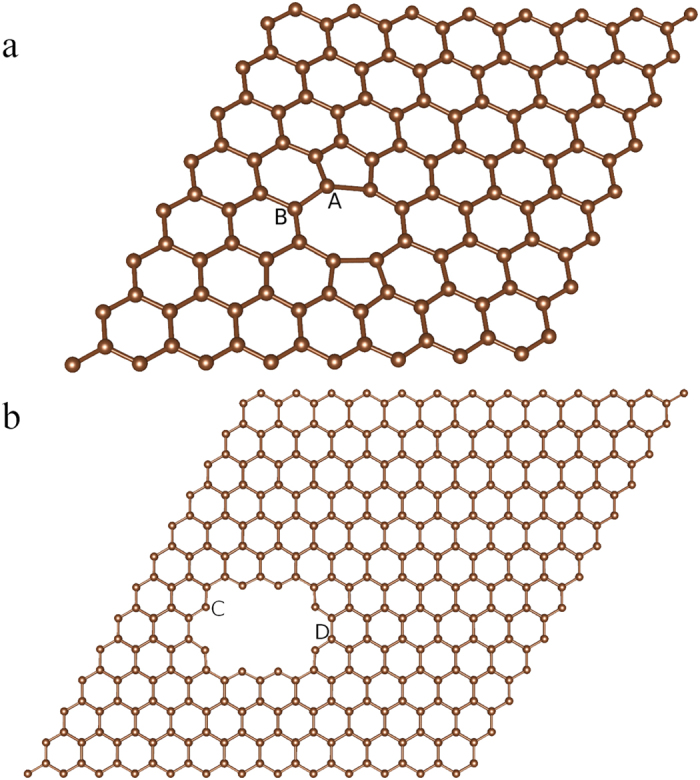
Ab initio density functional theory (DFT) calculation models of carbon-fluorine. Di-vacancy model (**a**) and hole-defect model (**b**), 0.95 nm in length, are based on the STM observation. Binding energies are shown in Table 1.

Ab initio density functional theory calculations were performed to investigate the adsorption behavior of fluorine atoms. In this calculation, two models, di-vacancy model and hole-defect model, are used as shown in Fig. 4a. These two types of defects were both observed in STM image at an ion dosage of 10^13^ ions/cm^2^. In these two models, there are four typical positions marked as site A-D. The calculated energetics shows that the adsorption energy (shown in Table 1) of fluorine on pristine graphene (−1.91 eV) is higher than the adsorption energy in di-vacancy (−2.86 eV at site A and −2.25 eV at site B, respectively) and hole-defect (−5.64 eV at site C and −2.18 eV at site D, respectively), meaning that the carbon atoms surrounding defects are more likely to react with F atoms. At site C, it is found that fluorine has significantly lower adsorption energy to carbon atom in radicalized state (dangling bond), and the new C-F bond only has one orientation that is in-plan, while C-F bonds in other sites are all out of plan (perpendicular to the graphene lattice). Even if we set the new C-F bond at site C is out of plane when forming, it will rotate to in-plan automatically. This in-plan C-F bond has a bond length of 0.136 nm, typical for sp^2^ hybridization, while others are all sp^3^ hybridization. This strong bond between dangling bond and saturated atoms implies that different gases could be utilized to functionalize graphene.

In conclusion, we have demonstrated a technique to realize site-selective local fluorination of graphene by utilizing high-energy ion irradiation and simultaneously XeF_2_ injection. It is found by X-ray photoelectron spectroscopy and scanning tunneling microscopy as well as from systematic fluorination experiments that the fluorine atoms are predominantly localized at the defect sites. This novel approach is based on the local radicalization of graphene that reacts with injected molecules and has the potential to functionalize graphene locally with a wide range of attached species.

## Methods

### Graphene sample preparation

Fluorination experiments were performed on large scale graphene synthesized by chemical vapor deposition (CVD) and transferred to SiO_2_/Si substrate (Graphene Supermarket, monolayer graphene on SiO_2_/Si wafer)^32^,^33^. Epitaxial graphene, prepared by SiC (0001) annealing in UHV^27^,^34^, was used for scanning tunneling microscopy (Omicron, LT-STM) observation to understand the atomic structure of irradiated graphene. From the Stopping and Range of Ions in Matter (SRIM) calculation, it is found that CVD graphene and epitaxial graphene have nearly the same concentration of vacancies (see Supporting Information S4). In contrast to CVD graphene on SiO_2_, epitaxial graphene on SiC has better surface quality and thus is suitable for STM imaging. However, SiC can react with injected XeF_2_ gas, making the analysis of graphene using XPS more ambiguous, while SiO_2_ substrate is more inert to fluorination^17^,^35^. Thus, CVD graphene was mainly used in fluorination experiment followed by spectroscopic analysis and epitaxial graphene was used for STM imaging.

### Characterizations

Before and after irradiation, Raman spectroscopy (Renishaw, inVia Raman with 532 nm excitation) and X-ray photoelectron spectroscopy (Quantum 2000 Scanning ESCA) spectra were taken to verify the formation of fluorinated graphene. Scanning tunneling microscopy (Omicron, LT-STM) was utilized to observe the atomic structure of irradiated graphene.

### Ab initio density functional theory (DFT)

Ab initio density functional theory calculations are performed by using a plane wave based pseudo potential method (VASP) to study the interactions between fluorine and carbon atoms^36^,^37^. The generalized gradient method of Perdew, Burke and Ernzerhof has been used for the exchange-correlation along with PAW potential^38^,^39^. Structures were optimized using conjugate gradient method with forces calculated from Hellman-Feynman theorem. The energy and force thresholds are kept at 10^−5^ eV and 10^−2^ eV/Å, respectively. For geometry optimization, a 5 × 5 × 1 Gamma centered **k** grid was used. For total energies and electronic structures, we have used an 11 × 11 × 1 Gamma centered **k** grid. The adsorption energy (*E*_*ads*_) of F atom adsorbed in graphene sheet is calculated using the following formula,


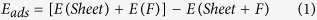


where *E*(*Sheet* + *F*) is the total energy of the F atom adsorbed in on the sheet, *E*(*Sheet*) is the total energy of the sheet without F atom and *E*(*F*) is the total energy of an isolated F atom kept in a box.

## Additional Information

**How to cite this article**: Li, H. *et al.* Site-selective local fluorination of graphene induced by focused ion beam irradiation. *Sci. Rep.* **6**, 19719; doi: 10.1038/srep19719 (2016).

## Supplementary Material

Supplementary Information

## Figures and Tables

**Table 1:** Adsorption energies of fluorine on pristine graphene as well as the edge carbon atoms surrounding defects.

Structure	E_abs_ (eV)	Hybridization
Pristine	−1.91	sp^3^
Di-vacancy site A	−2.86	sp^3^
Di-vacancy site B	−2.25	sp^3^
Hole-defect site C (dangling bond)	−5.64	sp^2^
Hole-defect site D	−2.18	sp^3^
