# Supplementary material for: Site-selective local fluorination of graphene induced by focused ion beam irradiation
Source: Sci Rep. 2016 Jan 29;6:19719. doi: 10.1038/srep19719 (PMC4731758; doi:10.1038/srep19719)
Supplement: Supplementary Information [file srep19719-s1.pdf]

Site-selective local fluorination of graphene induced by focused ion beam irradiation

Hu Li[1], Lakshya Daukiya[2], Soumyajyoti Haldar[3], Andreas Lindblad[4], Biplab Sanyal[3], Olle Eriksson[3], Dominique Aubel[2], Samar Hajjar-Garreau[2], Laurent Simon[2], and Klaus Leifer[1,*].

[1] Department of Engineering Sciences, Ångström Laboratory, Uppsala University, Sweden

[2] Institut de Sciences des Matériaux de Mulhouse, UMR 7361-CNRS, UHA, France

[3] Department of Physics and Astronomy, Ångström Laboratory, Uppsala University, Sweden

[4] Department of Chemistry, Ångström Laboratory, Uppsala University, Sweden

*Klaus.Leifer@angstrom.uu.se

S1. X-ray photoelectron spectroscopy (XPS) survey spectrum of fluorinated graphene (FG)

Ga^+ ions of 30 keV, utilized in experiments, have significant advantages compared with other ions. High energy Ga^+ ions can remove carbon atoms from graphene lattice, leading to the radicalization of carbon atoms surrounding defects. Then they propagate into the substrate and do thus not substitute carbon atoms nor accumulate on graphene surface. Figure S1 shows the XPS survey spectrum of fluorinated graphene (FG), and in this spectrum there is no Ga signal (binding energy at 1116.7 eV), which means that there is no Ga atoms on top of graphene.

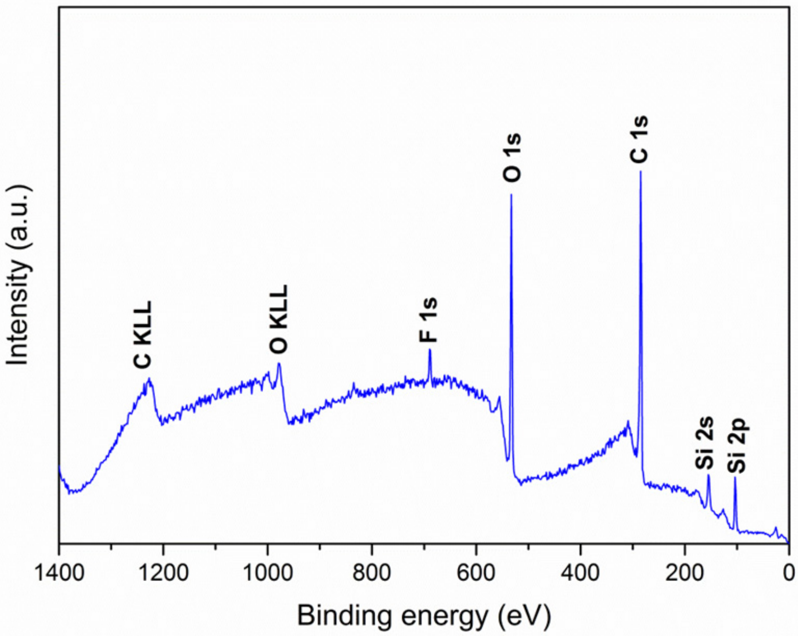

Figure S1. XPS survey spectrum of fluorinated graphene on SiO_2.

S2. Large scale of STM image of defected graphene (DG) under ion irradiation

Our DFT models, di-vacancy model and hole-defect model, are mainly based on the STM observation of graphene under ion irradiation. Figure S2 shows that, in large scale STM image of defected graphene (DG), the defects are mainly vacancies and small hole-defect. Similar defects are also found in fluorinated graphene (FG) shown in Figure 3.

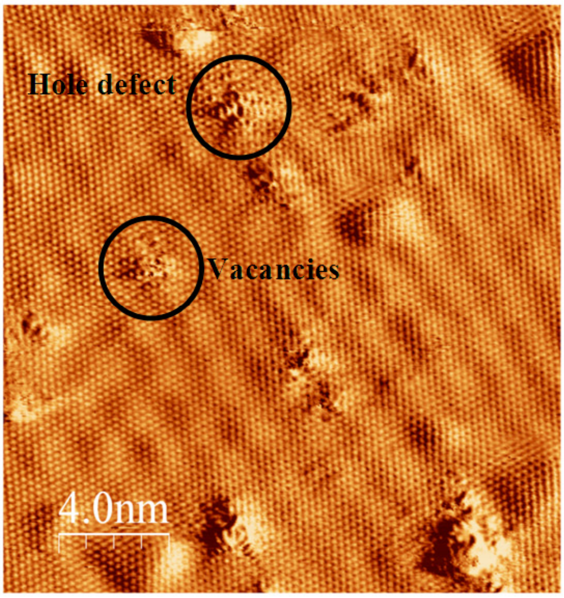

Figure S2. Large scale of derivative STM image of DG graphene

S3. XPS analysis of fluorinated graphene

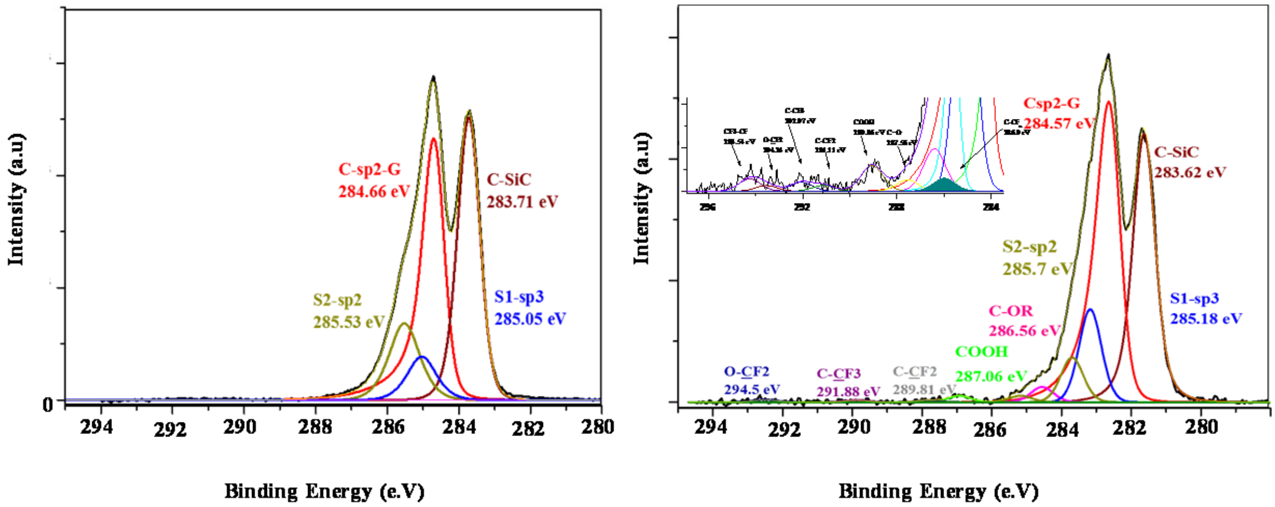

Figure S3. XPS analysis of C 1s peak before and after fluorination in SiC-graphene

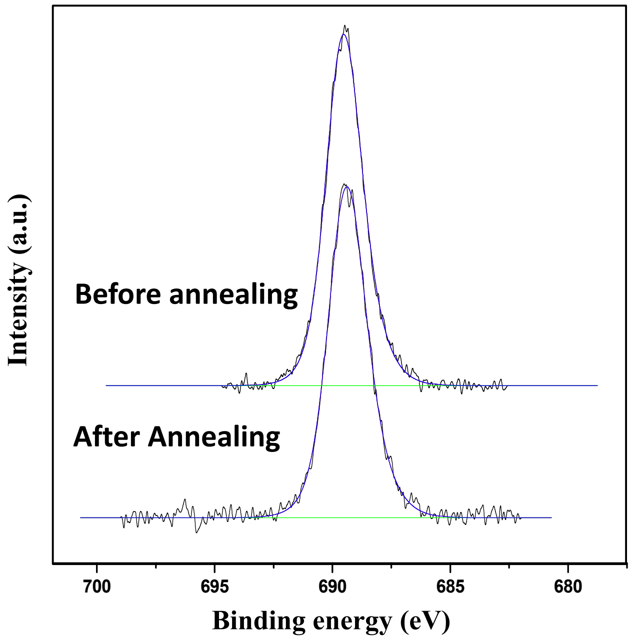

Figure S4. XPS analysis of F 1s peak of fluorinated graphene before and after annealing

Figure S3 shows the deconvolutions C 1s peak before and after fluorination, it is clear that there is a significant increase in C-F bond. To get clear STM images, a soft annealing was performed on fluorinated graphene with the temperature up to 200°C. Before and after annealing, it is found that the fluorine is stable.

S4. Monte Carlo Stopping and Range of Ions in Matter (SRIM) simulation

The sputtering simulation of 30 keV Ga^+ ion irradiation was performed by using Monte Carlo SRIM-2008. [1,2] Four samples are simulated: graphene on SiO_2 substrate (CVD graphene), graphene on SiC substrate (epitaxial graphene), SiO_2 and SiC.

Table S1. Sputtering yield of CVD graphene, epitaxial graphene, SiO_2 and SiC under 30 keV Ga^+ ion irradiation by Monte Carlo SRIM calculation.

	C atom	Si atom	O atom	Sputtering yield in total
Graphene on SiO_2	2	0.1	0.13	2.22
SiO_2		1	3.5	4.5
Graphene on SiC	2 (graphene) + 0.1 (SiC)	0.15		2.25
SiC	1.3	1.7		3

Table S1 shows the sputtering yields of four different samples. Comparing graphene on substrate with pure substrate, it is found that graphene layer has a significant reducing effect on the sputtering yield of substrate, meaning that in CVD graphene and epitaxial graphene, most sputtered atoms are graphene carbon atoms in contrast to substrate atoms.[3] Thus, the substrate only has small contribution to the total sputtering yield compared with graphene layer.

The sputtering yields of carbon and total atoms for CVD graphene and epitaxial graphene are nearly the same shown in Figure S4. Considering the low influence of the substrate on sputtering, same sputtering yield of carbon indicates the same concentration of defects density under a same ion dose irradiation. Thus, the structure of CVD graphene and epitaxial graphene should be comparable in our experiment under an ion irradiation of 10^{13} ions/cm^2.

S5. Graphene structure after adsorption of fluorine atoms

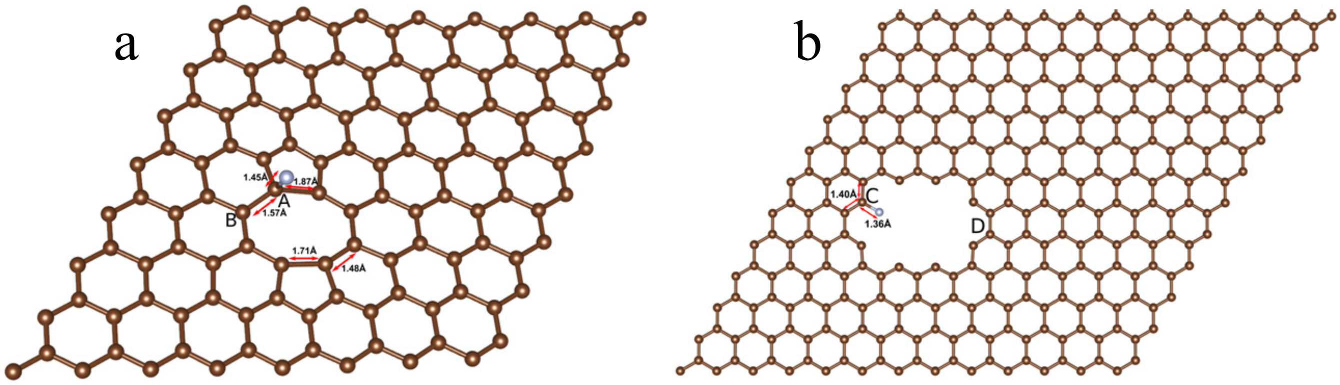

Figure S5. (a) graphene structure after adsorption at most probable site in di-vacancy defect. (b) graphene structure after adsorption at most probable site in a hole defect

Before the adsorption of F atom, the double vacancy creates a 585 (pentagon-octagon-pentagon) defect after geometry relaxation. The adsorption of F atom locally distorts the lattice shown in Figure S5 (a). The C atom attached to the F atom comes out of the graphene plane by 0.46 Å. Here C-F bond length is 1.45 Å and it forms a sp^3 bond. Distortions in the octagonal ring are also observed with different C-C bond lengths as shown in the figure. Figure S5 (b) indicates the structure after adsorption at most probable site in a hole defect. In this case, the F atom forms bond with the in-plane dangling bond of the C atom nearest to it. The C-F bond length is 1.36 Å and is of sp^2 type. The closest C-C bonds are shortened a bit (1.40 Å) as shown in the figure. In this case the structure is planar.

References:

1. Biersack, J. & Haggmark, L. A Monte Carlo computer program for the transport of energetic ions in amorphous targets. *Nucl. Instruments Methods* **174,** 257–269 (1980).

2. Robinson, M. & Torrens, I. Computer simulation of atomic-displacement cascades in solids in the binary-collision approximation. *Phys. Rev. B* **9,** (1974).

3. Lopez, J. J., Greer, F. & Greer, J. R. Enhanced resistance of single-layer graphene to ion bombardment. *J. Appl. Phys.* **107,** 104326 (2010).
